# Predictive factors of in-hospital mortality in patients with laboratory-confirmed *Escherichia coli*, *Klebsiella* species or *Pseudomonas aeruginosa* bloodstream infections

**DOI:** 10.1371/journal.pone.0259305

**Published:** 2021-11-02

**Authors:** Eleanor Mitchell, Mark Pearce, Anthony Roberts, Julia Newton

**Affiliations:** 1 Population Health Sciences Institute, Newcastle University, Newcastle upon Tyne, United Kingdom; 2 Academic Health Science Network – North East & North Cumbria, Newcastle upon Tyne, United Kingdom; 3 North East Quality Observatory Service (NEQOS), Newcastle upon Tyne, United Kingdom; 4 South Tees Hospitals NHS Foundation Trust, Middlesbrough, United Kingdom; Medical University of Gdansk, POLAND

## Abstract

Gram-negative bloodstream infections (GNBSI) are confirmed by the presence of gram-negative bacteria in the bloodstream and pose a significant healthcare issue as they increase the risk of sepsis and mortality. In England, the aim is to reduce GNBSI cases and further deterioration through enhanced population surveillance of patients with a laboratory-confirmed GNBSI to inform on healthcare policies. The objective of this study was to evaluate the factors associated with in-hospital mortality in patients with a laboratory-confirmed *Escherichia coli*, *Klebsiella* or *Pseudomonas aeruginosa* GNBSIs, with data obtained from the enhanced data capture for the surveillance of GNBSIs. All patients with a laboratory-confirmed GNBSI at a single centre, admitted between April 2017 and March 2019, were included in this retrospective observational study. Demographic and recent exposure to healthcare risk factors were collected and assessed for the association with in-hospital mortality. In 1113 patients with laboratory-confirmed GNBSIs, the in-hospital mortality rate was 13%. Multivariable analysis confirmed that patients with respiratory (OR = 3.73, 95%CI = 2.05–6.76), gastrointestinal (2.61; 1.22–5.58) or skin (3.61; 1.24–10.54) infection primary focus had a greater risk of in-hospital mortality, compared to upper urinary tract infections. Increased risk of in-hospital mortality was also observed in patients with hospital-onset GNBSIs (OR = 1.87; 1.17–2.97) compared with community-onset healthcare acquired GNBSIs, or who were on dialysis at the time of the GNBSI (3.28; 1.01–10.14), as well as in patients who had recently been discharged from hospital (1.55; 1.01–2.38), or had a vascular device recently manipulated (2.41; 1.01–5.74). Results confirm that the data obtained from the enhanced data capture for GNBSIs in England can predict in-hospital mortality in patients with a GNBSI. Several factors associated with an increased risk of in-hospital mortality have been identified. Results should be reported back to clinicians in order to identify patients at a greater risk of dying in-hospital who may benefit from further monitoring.

## Introduction

Gram-negative bloodstream infections (GNBSI) are defined by the presence of gram-negative bacteria within the bloodstream [1]. Approximately 70% of all GNBSIs in England are due to *Escherichia coli* (*E*. *coli*), *Klebsiella* and *Pseudomonas aeruginosa* (*P*. *aeruginosa*) species. National surveillance programmes have therefore been established in England to monitor these three main GNBSI associated species, with the aim to reduce rates of GNBSIs by 50% by 2024/5 [2, 3]. GNBSIs are a significant healthcare issue, as they are a leading cause of sepsis [1]. Sepsis is defined as a dysregulated host immune response to an infection and is a prominent healthcare issue, with 123,000 cases and 37,000 associated deaths each year [4–6]. As a result, clinicians aim to detect and treat GNBSIs that may result in sepsis quickly and effectively, to prevent the development of sepsis and further deterioration [7].

As part of the NHS Improvement programme to reduce GNBSIs, sepsis and the mortality associated, enhanced surveillance of GNBSIs began in England, in April 2017 [8]. Previous risk exposures and demographic data are routinely collected upon conformation of a laboratory-confirmed GNSBI and uploaded to a national database to monitor local rates and identify trends in infection and risks. At present, there are no studies that have investigated the use of this routinely collected data to predict in-hospital mortality in patients with a laboratory-confirmed GNBSI. Using this data could highlight areas of improvement and aid clinical decision making to identify patients at a greater risk of deterioration who would benefit from enhanced monitoring and targeted intervention.

## Methods

This retrospective observational study was conducted at South Tees Hospitals NHS Foundation Trust (STHFT), comprising of two acute hospitals and several community hospitals, located in the North East of England. Patients included in the study were admitted between 1st April 2017 and 31st March 2019 and included all cause first admissions only. All patients ≥18 years old with a laboratory-confirmed GNBSI, confirmed by the microbiology team at STHFT, were eligible. Positive GNBSIs were confirmed if one or more of *E*. *coli*, *Klebsiella* or *P*. *aeruginosa* species were present in the blood specimen. Enhanced surveillance data were collected by a member of the infection and prevention care team (IPCT) when confirmed, as GNBSI outlined by PHE [9, 10]. Additional patient data (admission method, admission discharge, admission date, postcode, primary diagnosis) was obtained through the STHFT Commissioning Data Set, whereby patient data were extracted through filters set by the user. Patient data were matched between the two datasets via patient hospital number and admission date.

Ethical approval for the study was granted by Newcastle University Ethics Committee, Ref: 10006/2018 (08/01/2019). Written consent to access and conduct research at STHFT was obtained from the STHFT Research & Development department and waived the requirement for informed patient consent (03/01/2019). Retrospective data collection and processing was carried out between July 2019 and December 2019.

### Data collection and definitions

Information from the PHE enhanced surveillance included in data analysis consisted of sex, age (set as a continuous variable for analysis), index of multiple deprivation (based on postcode and categorised from 1–10 with 1 being most deprived and 10 the least deprived) [11, 12], provenance (where the patient as located prior to hospital admission), episode (whether the GNBSI was a new, continuous or repeat infection), case definition grouped into COCA (Community onset community associated) determined if <2 days between admission and specimen date without discharge from STHFT in the previous 28 days, COHA (Community onset healthcare associated) determined if <2 days between admission and specimen date with discharge from STHFT in the previous 28 days, or HOHA (Hospital onset healthcare associated) determined if >2 days between admission and specimen date [9, 10].

Information on the primary focus of the GNBSI were also collected. This consisted of where the infection is thought to have originated from, including an ‘Unknown’ primary focus if not known. Risk factors known to be associated with healthcare interactions were also obtained, with the following variables assessed for the occurrence within the 28 days prior to the GNBSI episode; urinary catheter in place, urinary catheter manipulated, prostate biopsy, vascular access device (PPM, ICD or CVC manipulated), urinary tract infection treatment, intubated (ET or PT) or extubated, surgery prior to procedure (30 days or 12 months prosthetic material), hepatobiliary procedure (ERCP or MRCP), open wounds/ulcer not diabetic foot infection, diabetic foot infection or ulcer. Absolute neutrophil count (<500 cells/μL) at the time of the GNBSI episode and whether the patient was on dialysis at the time of the GNBSI was also collected. Patients were categorised into having a GNBSI with sepsis or GNBSI without sepsis, dependent on the presence of a sepsis ICD-10 code (A40/A41) in the primary diagnosis position [13, 14].

### Statistical analysis

All analysis was carried out on STATA Version 14.0 (StataCorp; College Station, TX, USA). Development of the multivariable model was in line with the recommendations stated by the Transparent reporting of a multivariable prediction model for individual prognosis or diagnosis (TRIPOD) [15]. Complete case analysis was carried out, meaning that any observation with a missing value for any variable was discarded from analysis.

The outcome of interest in this study was in-hospital mortality. This was identified through patient discharge method, “4: Patient Died”. The association between categorical data and in-hospital mortality was tested using Pearson’s chi-squared test, Fisher’s exact test or Mann-Whitney U tests, were appropriate. Variables with a relaxed p-value (p<0.25) in univariate analysis were considered for inclusion in the multivariable logistic regression model. Multicollinearity between variables included in the multivariable model were tested by examining the variance inflation factor (VIF). VIF >10 was indicative of multicollinearity.

Forward selection was used to create the multivariable logistic regression model. Variables were added into the model one at a time, beginning with the most significant variable from univariate analysis. A likelihood ratio test with p<0.15 was used as the threshold for inclusion in the model. The process was repeated until no other variables significantly improved the model. Potential confounders identified from the literature were also added into the multivariable model. The Hosmer-Lemeshow goodness of fit test determined whether the model was an overall good fit for predicting in-hospital mortality. Potential confounding variables were tested by a change in estimate (CIE), with ≥10% change used as the threshold to consider a variable a potential confounder when removed from the multivariable model [16]. All relevant interaction terms were tested. If any interaction terms significantly improved the model, determined by the likelihood ratio test which is the statistical test of the goodness-of-fit between two models, further investigation was carried out, including assess interaction on additive and multiplicative scales as previously recommended [17]. P-values <0.05 were considered significant.

## Results

### Patient characteristics

A total of 1113 patients with a laboratory-confirmed GNBSI were admitted to STHFT between April 2017 and March 2019. Overall, there were more patients coded GNBSI without sepsis (61.01%), a greater proportion of patients were male (52.7%), and the median age was 74 (18–100). There was very limited missing data, attributed to by the mandatory aspect of the PHE enhanced data collection. Overall, 6 patients were excluded due to missing data for several variables.

There were 153 (13.75%) in-hospital deaths during the study period. Patients who died were significantly older males, more likely to have been in-hospital prior to the GNBSI episode, more likely to have a HOHA infection and more likely to have a respiratory infection as the primary focus. 24.84% of those who died had been on dialysis at the time of the GNBSI, 9.15% had had a vascular device in situ, or manipulated within 28 days prior to the GNBSI, and 7.84% had an open wound in the 28 days prior to the GNBSI. Over a quarter of patients with a GNBSI who died in-hospital were categorised as having an IMD of 1. The fewest number of deaths were reported in patients who had an IMD score of 10 (3.27%). *E*. *coli* infections accounted for 75.65% of cases overall. In patients with a GNBSI who died in-hospital, the majority had an *E*. *coli* infection. The next most common infection was due to *Klebsiella* species, followed by *P*. *aeruginosa* (Table 1).

**Table 1:** Demographic factors associated with in-hospital mortality in patients with GNBSI (n = 1113).

Patient characteristics		Total (%) n = 1113	Survivors (%) n = 960 (86.25)	Non survivors (%) n = 153 (13.75)	*p*-value
GNBSI with sepsis		434 (38.99)	376 (39.17)	58 (37.91)	0.767
GNBSI without sepsis		679 (61.01)	584 (60.83)	95 (62.09)	
Sex	Male	587 (52.74)	495 (51.56)	92 (60.13)	0.049
Female		526 (47.26)	465 (48.44)	61 (39.87)	
Age		74 (63–83) ^¥^			<0.001*
<50		103 (9.25)	100 (10.42)	3 (1.96)	
50–59		112 (10.06)	100 (10.42)	12 (7.84)	
60–69		208 (18.69)	186 (19.38)	22 (14.38)	
70–79		297 (26.68)	250 (26.04)	47 (30.72)	
80–89		305 (27.40)	251 (26.15)	54 (35.29)	
90+		88 (7.91)	73 (7.60)	15 (9.80)	
IMD		4 (1–7) ^¥^			0.813*
1		302 (27.13)	260 (27.08)	42 (27.45)	
2		93 (8.36)	85 (8.85)	8 (5.23)	
3		104 (9.34)	85 (8.85)	19 (12.42)	
4		102 (9.16)	85 (8.85)	17 (11.11)	
5		93 (8.36)	82 (8.54)	11 (7.19)	
6		72 (6.47)	60 (6.25)	12 (7.84)	
7		100 (8.98)	88 (9.17)	12 (7.84)	
8		122 (10.96)	107 (11.15)	15 (9.80)	
9		73 (6.56)	61 (6.35)	12 (7.84)	
10		52 (4.67)	47 (4.90)	5 (3.27)	
Provenance	Home	946 (85.00)	828 (86.25)	118 (77.12)	0.001
Nursing		141 (12.67)	115 (11.98)	26 (16.99)	
Hospital		26 (2.34)	17 (1.77)	9 (5.88)	
Case definition	COCA	290 (26.06)	256 (26.67)	34 (22.22)	<0.001
COHA		601 (54.00)	532 (55.42)	69 (45.10)	
HOHA		222 (19.95)	172 (17.92)	50 (32.68)	
Episode	New	1058 (95.06)	915 (95.31)	143 (93.46)	0.260 Ϯ
Relapse		9 (0.81)	9 (0.94)	0 (0)	
Continuing		31 (2.79)	24 (2.50)	7 (4.58)	
Unknown		15 (1.35)	12 (1.25)	3 (1.96)	
Bacteria species	*P*. *aeruginosa*	53 (4.76)	40 (4.17)	13 (8.50)	0.075
*Klebsiella*		184 (16.53)	156 (16.25)	28 (18.30)	
*E*. *coli*		842 (75.65)	736 (76.67)	106 (69.28)	
Multibacteraemia		34 (3.05)	28 (2.92)	6 (3.92)	
Admission Method	Elective	59 (5.30)	52 (5.42)	7 (4.58)	0.699
A&E		1054 (94.70)	908 (94.58)	146 (95.42)	
Patient Category	In-patient	971 (87.24)	838 (87.29)	133 (86.93)	0.900
A&E		142 (12.76)	122 (12.71)	20 (13.07)	
Primary focus	Gastrointestinal	62 (5.57)	50 (5.21)	12 (7.84)	<0.001
Hepatobiliary		153 (13.75)	132 (13.75)	21 (13.73)	
Skin		23 (2.07)	16 (1.67)	7 (4.58)	
LUTI		369 (33.15)	332 (34.58)	37 (24.18)	
UUTI		147 (13.21)	135 (14.06)	12 (7.84)	
Respiratory		90 (8.09)	64 (6.67)	26 (16.99)	
IVD		14 (1.26)	13 (1.35)	1 (0.65)	
No focus		35 (3.14)	33 (3.44)	2 (1.31)	
Unknown		209 (18.78)	177 (18.44)	32 (20.92)	
Other		11 (0.99)	8 (0.83)	3 (1.96)	
HCA risk factor present	No	475 (42.68)	416 (43.33)	59 (38.56)	0.268
Yes		638 (57.32)	544 (56.67)	94 (61.44)	
Discharge	No	887 (79.69)	772 (80.42)	115 (75.16)	0.134
Yes		226 (20.31)	188 (19.58)	38 (24.84)	
Dialysis	No	1095 (98.38)	949 (98.85)	146 (95.42)	0.002
Yes		18 (1.62)	11 (1.15)	7 (4.58)	
Neutrophil <500 cells/μL	No	1037 (93.17)	897 (93.44)	140 (91.50)	0.378
Yes		76 (6.83)	63 (6.56)	13 (8.50)	
Diabetic foot	No	1103 (99.10)	953 (99.27)	150 (98.04)	0.148 Ϯ
Yes		10 (0.90)	7 (0.73)	3 (1.96)	
Hepatobiliary procedure	No	1096 (98.47)	946 (98.54)	150 (98.04)	0.719 Ϯ
Yes		17 (1.53)	14 (1.46)	3 (1.96)	
Intubated	No	1074 (96.50)	931 (96.98)	143 (93.46)	0.028
Yes		39 (3.50)	29 (3.02)	10 (6.54)	
Open wounds	No	1067 (95.87)	9.26 (96.46)	141 (92.16)	0.013
Yes		46 (4.13)	34 (3.54)	12 (7.84)	
Surgery	No	958 (86.07)	827 (86.15)	131 (85.62)	0.862
Yes		155 (13.93)	133 (13.85)	22 (14.38)	
Vascular device	No	1051 (94.43)	912 (95.00)	139 (90.85)	0.038
Yes		62 (5.57)	48 (5.00)	14 (9.15)	
Catheter	No	888 (79.78)	768 (80.00)	120 (78.43)	0.654
Yes		225 (20.22)	192 (20.00)	33 (21.57)	
Catheter manipulated	No	913 (82.03)	790 (82.29)	123 (80.39)	0.570
Yes		200 (17.97)	170 (17.71)	30 (19.61)	
UTI treatment	No	962 (86.43)	823 (85.73)	139 (90.85)	0.086
Yes		151 (13.57)	137 (14.27)	14 (9.15)	
Antibiotics	No	715 (64.24)	625 (65.10)	90 (58.82)	
Yes		398 (35.76)	335 (34.90)	63 (41.18)	0.132

IMD; Index of Multiple Deprivation, COCA; Community-onset community associated, COHA; Community-onset healthcare associated, HOHA; Hospital-onset healthcare associated, *P*. *aeruginosa*; *Pseudomonas aeruginosa*, *E*. *coli*; *Escherichia coli*, A&E; Accident and emergency LUTI; Lower urinary tract infection, UUTI; Upper urinary tract infection, IVD; Intravascular device UTI; Urinary tract infection.

^¥^Median (Inter-quartile range),

* Mann-Whitney U test,

^Ϯ^ Fisher’s exact test.

Overall, 58 (37.91%) patients who died had a sepsis ICD-10 primary diagnosis code and 95 (62.09%) patients were without a sepsis primary diagnosis ICD-10 code. There was no significant difference in mortality rates in GNBSI patients with and without a sepsis primary diagnosis (Table 1). Of the A40/41 codes in the primary diagnosis position, A41.5 (sepsis due to other gram-negative organisms) was the most commonly observed in patients admitted (76%) and who died (62%) in-hospital. Of patients without a sepsis primary diagnosis code, the most common group of ICD-10 codes were genitourinary codes (31%), followed by digestive disorder codes (18%). The most common codes in patients who died included respiratory (22%) then neoplasms (21%) (Table 2).

**Table 2:** Most commonly observed ICD-10 code categories in patients with a GNBSI with sepsis and GNBSI without sepsis (n = 1113).

	Most common sepsis ICD-10 codes	Most common sepsis ICD-10 codes in patients who died in-hospital	Most common non-sepsis ICD-10 codes	Most common non-sepsis ICD-10 codes in patients who died in-hospital
1.	Gram-negative sepsis (76%)	Gram-negative sepsis (62%)	Genitourinary (31%)	Respiratory (22%)
2.	Sepsis unspecified (18%)	Sepsis unspecified (34%)	Digestive (18%)	Neoplasms (21%)
3.	Sepsis other specified (4%)	Sepsis other specified (3%)	Other (13%)	Digestive (16%)
4.	-	-	Respiratory (11%)	Circulatory (16%)
5.	-	-	Neoplasms (10%)	Genitourinary (14%)

ICD-10; International Classification of Diseases—Tenth Edition.

### Multivariable logistic regression models

The independent predictors of in-hospital mortality in patients with a GNBSI are presented in Table 3, from univariate and multivariable analysis. The test for multicollinearity found no correlation between variables in the regression model (VIF<10) (S2 Table). The adjusted multivariable logistic regression model had an adequate goodness to fit (Hosmer-Lemeshow = 7.17, p = 0.519). GNBSIs with a gastrointestinal (OR = 2.61, 95% CI = 1.22–5.58), skin (OR = 3.61, 95% CI = 1.24–10.54) or respiratory (OR = 3.73, 95%CI = 2.05–6.76) primary focus were independently associated with an increased risk of in-hospital mortality. Age was significantly associated with in-hospital mortality, with odds increasing by a factor of 1.04 (95% CI = 1.02–1.05) per each increasing year of age. No significant association was observed for location of the patient prior to the GNBSI episode. HOHA infections were associated with an increased risk of in-hospital mortality in contrast with the reference category, COHA (OR = 1.87, 95% CI = 1.17–2.97). The risk of in-hospital mortality was also greater in patients who were on dialysis at the time of the GNBSI (OR = 3.28, 95% CI = 1.06–10.14). In addition, odds of in-hospital mortality were 2.41 (95% CI = 1.01–5.74) and 1.55 (95% CI = 1.01–2.38) higher in patients who had a vascular device or were discharged from hospital within the 28 days prior to the GNBSI, respectively. The potential confounding variables sex, IMD and neutrophil count <500 cells/μL at the time of the GNSBI did not result in a CIE ≥10% of odds ratios when removed from the fully adjusted model (S1 Table). Removing age from the model changed the odds ratio for the risk of in-hospital mortality for the variables skin primary focus (24%), patients residing in a nursing home prior to hospitalisation (39%), being on dialysis at the time of the GNBSI (12%) and having a recent vascular device (15%) (S1 Table). The changes in the ORs once age is removed suggests that age is a confounder, and it is necessary to adjust for age to ensure the true association of each variable on the risk of in-hospital mortality.

**Table 3:** Univariate and multivariable logistic regression for the explanatory variables for the prediction of in-hospital mortality in patients with GNBSI (n = 1113).

In-hospital mortality	Unadjusted Odds Ratio	95% CI	*p*-value	Adjusted Odds Ratio	95% CI	*p*-value
Primary focus						<0.001
Gastrointestinal	2.15	1.05–4.41	*0*.*036*	2.61	1.22–5.58	*0*.*013*
Hepatobiliary	1.42	0.81–2.52	*0*.*223*	1.54	0.85–2.79	*0*.*150*
Skin	3.92	1.51–10.16	*0*.*005*	3.61	1.24–10.54	*0*.*019*
LUTI	Ref.	-	*-*	Ref.	-	*-*
UUTI	0.79	0.4–1.57	*0*.*515*	0.99	0.49–2.01	*0*.*979*
Respiratory	3.64	2.06–6.43	*<0*.*001*	3.73	2.05–6.76	*<0*.*001*
IVD	0.69	0.08–5.42	*0*.*725*	0.32	0.03–3.24	*0*.*335*
no focus	0.54	0.12–2.35	*0*.*416*	0.59	0.13–2.65	*0*.*489*
Unknown	1.62	0.97–2.69	*0*.*061*	1.38	0.76–2.53	*0*.*294*
Other	3.36	0.85–13.23	*0*.*083*	5.00	1.13–22.06	*0*.*054*
Age (years)	1.03	1.02–1.04	*<0*.*001*	1.04	1.02–1.05	<0.001
Case definition						0.001
COCA	1.02	0.66–1.59	*0*.*915*	0.99	0.62–1.59	*0*.*974*
COHA	Ref.	-	*-*	Ref.	-	*-*
HOHA	2.24	1.50–3.35	*<0*.*001*	1.87	1.17–2.97	*0*.*009*
Provenance						0.022
Home	Ref.	-	*-*	Ref.	-	*-*
Nursing	1.59	0.99–2.53	*0*.*053*	1.59	0.94–2.69	*0*.*083*
Hospital	3.71	1.62–8.52	*0*.*002*	2.46	0.94–6.43	*0*.*065*
On dialysis +						0.033
No	Ref.	-	*-*	Ref.	-	*-*
Yes	4.14	1.58–10.84	*0*.*004*	3.28	1.06–10.14	*0*.*040*
Vascular device <28 days*						0.033
No	Ref.	-	*-*	Ref.	-	*-*
Yes	1.91	1.03–3.56	*0*.*041*	2.41	1.01–5.74	*0*.*047*
Discharge <28 days *						0.048
No	Ref.	-	*-*	Ref.	-	*-*
Yes	1.36	0.91–2.02	*0*.*135*	1.55	1.01–2.38	*0*.*047*
Sex						0.218
Male	Ref.	-	*-*	Ref.	-	*-*
Female	0.71	0.50–0.99	*0*.*049*	0.80	0.55–1.15	*0*.*227*
IMD	0.99	0.94–1.05	*0*.*780*	0.98	0.92–1.04	0.520
Neutrophil <500 cells/μL +						0.255
No	Ref.	-	*-*	Ref.	-	*-*
Yes	1.32	0.71–2.47	*0*.*380*	1.48	0.75–2.91	*0*.*257*

LUTI; Lower urinary tract infection, UUTI; Upper urinary tract infection, IVD; Intravascular device, COCA; Community-onset community associated, COHA; Community-onset healthcare associated, HOHA; Hospital-onset healthcare associated, IMD; Index of Multiple Deprivation,

^+^ at the time of the GNBSI,

*<28 days prior to the GNBSI.

Investigations into interaction terms found that there was a significant positive interaction on the additive and multiplicative scale between recent hospital discharge and vascular device, indicating that patients with both risk factors have an increased risk of in-hospital mortality (p = 0.030). Table 4 shows the association between a recent hospital discharge and in-hospital mortality for patients with and without a recent vascular device.

**Table 4:** Recent vascular device and recent hospital discharge interaction for in-hospital mortality (n = 1113).

	Recent vascular device					
	No			Yes		
	Cases/subjects	OR (95% CI)	*p*	Cases/subjects	OR (95% CI)	*p*
Recent hospital discharge						
No	107/734	Ref.		8/38	1.50 (0.55–4.10)	0.431
Yes	32/178	1.36 (0.86–2.14)	0.188	6/10	5.90 (1.18–29.47)	**0.030**

Bold indicates significant interaction (p<0.05). Adjusted for primary focus, age, case definition, vascular device, discharge <28 days prior to GNBSI, sex, IMD and neutrophil count <500 cells/μL at the time of the GNBSI.

Measure of interaction on additive scale: RERIOR = 4.04 (5.90–1.50–1.36 + 1).

Measure of interaction on multiplicative scale = 2.89 (5.90 / (1.50 x 1.36).

In addition, there was a significant negative interaction on the additive and multiplicative scale between patients who had a recent vascular device and HOHA GNBSIs, indicating reduced risk of in-hospital mortality in patients with both variables (p = 0.020). The association between HOHA GNBSIs on the risk of in-hospital mortality in patients with and without a recent vascular device is shown in Table 5.

**Table 5:** Recent vascular device and case definition interaction for in-hospital mortality (n = 1113).

	Recent vascular device					
	No			Yes		
	Cases/subjects	OR (95% CI)	*p*	Cases/subjects	OR (95% CI)	*p*
Case definition						
COCA	32/252	0.99 (0.61–1.62)	0.988	2/4	2.05 (0.19–21.83)	0.553
COHA	63/512	Ref.		6/20	5.61 (1.80–17.78)	0.003
HOHA	44/148	2.22 (1.37–3.60)	0.001	6/24	0.17 (0.04–0.76)	**0.020**

Bold indicates significant interaction (p<0.05). Adjusted for primary focus, age, case definition, vascular device, discharge <28 days prior to GNBSI, sex, IMD and neutrophil count <500 cells/μL at the time of the GNBSI.

Measure of interaction on additive scale (COCAxRecent vascular device): RERIOR = -3.55 (2.05–5.61–0.99 + 1), Measure of interaction on additive scale (HOHAxRecent vascular device): RERIOR = -6.66 (0.17–5.61–2.22 + 1).

Measure of interaction on multiplicative scale (COCAxRecent vascular device) = 0.37 (2.05 / (0.99 x 5.61).

Measure of interaction on multiplicative scale (HOHAxRecent vascular device) = 0.01 (0.17 / (2.22 x 5.61).

## Discussion

In this retrospective observational study of patients with a laboratory-confirmed GNBSI admitted to STHFT, it was observed that patients with HOHA infections, respiratory, skin and gastrointestinal infections leading to the GNBSI, being on dialysis at the time of the GNBSI, having a recent hospital discharge or a recent vascular device were at significant increased risk of in-hospital mortality. To the best of our knowledge, this study is the first to use the national enhanced surveillance data for GNBSIs to predict in-hospital mortality. These results indicate that there are several characteristics of patients with a GNBSI that increase the risk of in-hospital mortality, highlighting a subgroup of patients who may benefit from more frequent monitoring to earlier identify deterioration.

In this study, the odds of in-hospital mortality were 3.73 times greater in patients with a respiratory primary focus compared to a LUTI primary focus. It has been reported previously that respiratory infections leading to a BSI have poorer outcomes, whereas urinary tract infections have the lowest associated mortality rates [7, 18–22]. The recent study by Inada-Kim et. al assessed the most common diseases associated with suspicion of sepsis (SOS) codes and early antibiotic requirement. Respiratory diseases accounted for 39% of the SOS codes and 69.8% of patients with an SOS code who died in-hospital [7]. In agreement with results from the present study, Klastrup et al. found that when compared to sepsis patients with a gastrointestinal infections site, urinary tract infections had a significantly reduced risk of 30-day morality (OR = 0.34) [23]. It has previously been identified that both *P*. *aeruginosa* and *Klebsiella* bacteria species are more common in respiratory infections, and are associated with poorer outcomes, whereas *E*. *coli* infections are more common in UTI infections with favourable outcomes [2, 18, 24]. In this study, bacteria species type did not reach the significance threshold for inclusion in the multivariable model and so the independent association with in-hospital mortality was not assessed in the multivariable logistic regression model nor was the variable included in the interaction analysis.

Interestingly results in this study showed no significant difference in mortality rates between GNBSI patients with and without a sepsis primary diagnosis, suggesting that the development of sepsis was not an effect modifier in the association between GNBSI and in-hospital mortality. This observation is in contrast to the theory by Huerta et al., that discussed the pathological differences between BSIs and sepsis, and confirmed that sepsis is the more severe illness [1, 5]. It is interesting to postulate whether there are baseline differences in terms of demographic and healthcare-associated risk factors between GNBSI patients with and without a sepsis primary diagnosis and whether these impact on clinical care and management, and the subsequent risk of in-hospital mortality. For example, in England, the NICE guidelines are followed upon the suspicion of sepsis, which contain the recommendations to immediately start empirical antibiotic treatment, known to improve patient outcomes. Future studies that focus on the differences in baseline factors and time to effective treatment, between GNSBI patients with and without a sepsis primary diagnosis, would add value to this research field and help investigate why patients who have a less severe acute illness (GNBSI without a sepsis primary diagnosis) have a similar mortality rate as GNBSI with a sepsis primary diagnosis.

Several studies have observed higher mortality rates in patients with hospital acquired infections, similar to the observation in this study [24–27]. Previous studies have confirmed that HOHA infections are affiliated with patients who are more acutely ill, with HOHA-associated sepsis remaining independently associated with mortality (OR = 2.1), increased length of stay (19 v 8 days) and more ICU admissions compared with community-onset sepsis after adjustment for baseline illness [26]. Results concur with the theory that second hit infections, described as acquiring an infection whilst already in-hospital, contribute to a greater number of in-hospital deaths compared with primary hit infections [28].

Over 20% of patients included in the present study had been discharged from hospital within the 28 days prior to the GNBSI, and this was independently associated with a 1.55 increased odds in the risk of in-hospital mortality, consistent with a recent large study [8, 29]. In agreement with the findings in this study, a large retrospective cohort study of patients who were readmitted to hospital, established that readmission was associated with an increased risk of mortality, in addition to increased age, comorbidities, emergency admission [30]. In the patients who died, infections were the primary cause of readmission. A large, recent systematic review also found that in sepsis survivors, rehospitalisation was common, with infections being the most common cause of readmission [31]. Predictors found to be associated with rehospitalisation were also identified as risk factors for mortality in sepsis patients, including older age, male sex, comorbidities, site of infection and lower socioeconomic status. As this present study focused on first admissions of patients during the study period, future studies should focus on patients with multiple admissions to identify patients more likely to be readmitted and deteriorate.

Some studies have reported a significant association between socioeconomic status and mortality, with others finding no significant association [32–34]. IMD was not reported to be significantly associated with in-hospital mortality in either univariate or multivariable analysis in this study. Gharbi et al. found that in patients with a community onset UTI, older males living in more deprived areas were at a greater risk of developing sepsis and dying within 60-days of the UTI [33]. Whereas a large study in the US found patients with a lower socioeconomic status had a greater risk of infection and hospitalisation, but no difference was found in the risk of sepsis between patients from high and lower socioeconomic groups [32]. It should be noted that a greater proportion (35.5%) of patients included in this study were from the two most deprived IMD categories, compared to patients in the Gharbi study, which may have affected observed associations. The North East of England has some of the most deprived areas in England, so results in this study may not be replicated in-hospitals treating patients living in less deprived areas.

Similar to previous studies, patients with a recent vascular device present or manipulated and patients who were on dialysis at the time of the GNBSI were at a significantly greater risk of in-hospital mortality [35–38]. This study also identified a significant interaction between patients having a recent vascular device and recent hospital discharge on the risk of in-hospital mortality. Central venous catheter (CVC)-BSIs have been confirmed in several studies to be risk factors for mortality, attributed to by the repeated need for vascular access addition to patients more likely to have a poorer baseline health status, lending to a greater risk of deterioration [39]. Aminzadeh et al. reported that CVC-BSIs constituted as a large proportion of high-risk patients in non-ICU settings, with the first two weeks after CVC insertion having the highest risk of developing a BSI and 69.2% of CVC-BSIs occurred within <4 weeks of line insertion [40].

Significant negative interactions showed that patients with HOHA GNBSIs and a recent vascular device had a reduced risk of in-hospital mortality. It should be noted however, there was no significant difference between patients with HOHA GNBSI and a recent vascular device, compared to patients with COCA GNBSI and a recent vascular device, as there was an overlap in the 95% confidence intervals. This observation, and the significant positive interaction between patients with a recent vascular device and recent hospital discharge, should be interpreted with caution due to the small numbers of patients in analysis and wide confidence intervals. Therefore, larger studies are required to confirm the observations of this study.

There were several limitations within this study. Firstly, it was a single retrospective observational study meaning that it is not possible to establish causal effects. However, all possible confounders identified from previous studies were adjusted for in analysis to reduce the likelihood of the observations being due to the influence of confounding variables. Residual confounding may still be present however, as there are no clinical measurements included in analysis, such as comorbidity scores or illness severity measurements used to generate currently used risk prediction scores. In addition, information relating to source control, resistance patterns of bacterium species or time to appropriate antibiotics was not available. As all have been previously associated with in-hospital mortality in patients with BSIs or sepsis, it is likely that this would have impacted on observations [41–43].

Secondly, as the patients included in analyses were admitted to one hospital trust, results cannot be representative of the general population. Larger nationwide studies are required in the future to validate the observations in this study. Although the three gram-negative bacteria species included in this study account for 70% of all GNBSIs in England, data from patients with other gram-negative bacteria species were not collected or included in analysis. As a result, observations in this study are only representative of GNBSIs caused by *E*. *coli*, *Klebsiella* or *P*. *aeruginosa* species.

To conclude, this study shows that HOHA infections, respiratory, skin and gastrointestinal infections leading to the GNBSI, being on dialysis at the time of the GNBSI, having a recent hospital discharge or a recent vascular device were at significant increased risk of in-hospital mortality in patients with a laboratory-confirmed GNBSI. Results from this study may inform on clinical practice at STHFT as they highlight possible areas of improvement, focused around reducing HOHA infections and infections associated with dialysis and vascular devices. Furthermore, with completion of larger-scale, multicentre studies that validate the results of this study, a risk prediction model for patients with laboratory-confirmed GNBSI could be developed in order to assess the risk of mortality within hospital settings.

## Supporting information

S1 Table Change in estimates with removal of potential confounding variables from the fully adjusted model. CIE ≥10% in bold. LUTI; Lower urinary tract infection, UUTI; Upper urinary tract infection, IVD; Intravascular device, COCA; Community-onset community associated, COHA; Community-onset healthcare associated, HOHA; Hospital-onset healthcare associated IMD; Index of Multiple Deprivation, + At the time of the GNBSI, * Within <28 days prior to the GNBSI.
(DOCX)

S2 Table Test of multicollinearity of variables included in the multivariate logistic regression model. VIF; Variance inflation factor, LUTI; Lower urinary tract infection, UUTI; Upper urinary tract infection, IVD; Intravascular device, COCA; Community-onset community associated, COHA; Community-onset healthcare associated, HOHA; Hospital-onset healthcare associated IMD; Index of Multiple Deprivation, + At the time of the GNBSI, * Within <28 days prior to the GNBSI.
(DOCX)
