# Supplementary material for: Predictive factors of in-hospital mortality in patients with laboratory-confirmed Escherichia coli, Klebsiella species or Pseudomonas aeruginosa bloodstream infections
Source: PLoS One. 2021 Nov 2;16(11):e0259305. doi: 10.1371/journal.pone.0259305 (PMC8562814; doi:10.1371/journal.pone.0259305)
Supplement: S1 Table — CIE ≥10% in bold. LUTI; Lower urinary tract infection, UUTI; Upper urinary tract infection, IVD; Intravascular device, COCA; Community-onset community associated, COHA; Community-onset healthcare associated, HOHA; Hospital-onset healthcare associated IMD; Index of Multiple Deprivation, + At the time of the GNBSI, * Within <28 days prior to the GNBSI. (DOCX) [file pone.0259305.s001.docx]

**S1 Table**

|  | **Fully adjusted model** | | | **Removal of Age** | | | | **Removal of Sex** | | | | **Removal of IMD** | | | | **Removal of Neutrophil count +** | | | |
| --- | --- | --- | --- | --- | --- | --- | --- | --- | --- | --- | --- | --- | --- | --- | --- | --- | --- | --- | --- |
|  | **OR** | **95% CI** | ***p*** | **OR** | **95% CI** | ***p*** | **CIE %** | **OR** | **95% CI** | ***p*** | **CIE %** | **OR** | **95% CI** | ***p*** | **CIE %** | **OR** | **95% CI** | ***p*** | **CIE %** |
| Primary focus | |  |  |  |  |  |  |  |  |  |  |  |  |  |  |  |  |  |  |
| Gastrointestinal | 2.61 | 1.22-5.58 | 0.013 | 2.41 | 1.14-5.09 | 0.021 | -7.66 | 2.58 | 1.21-5.52 | 0.014 | -1.15 | 2.60 | 1.21-5.56 | 0.014 | -0.38 | 2.60 | 1.21-5.55 | 0.014 | -0.38 |
| Hepatology | 1.54 | 0.85-2.79 | 0.150 | 1.61 | 0.89-2.90 | 0.114 | 4.55 | 1.56 | 0.86-2.82 | 0.141 | 1.30 | 1.53 | 0.85-2.77 | 0.156 | -0.65 | 1.55 | 0.86-2.79 | 0.148 | 0.65 |
| Skin | 3.61 | 1.24-10.54 | 0.019 | 2.74 | 0.97-7.74 | 0.057 | **-24.10** | 3.61 | 1.24-10.52 | 0.019 | 0.00 | 3.61 | 1.24-10.52 | 0.019 | 0.00 | 3.57 | 1.23-10.38 | 0.019 | -1.11 |
| LUTI | Ref. | - | - | Ref. | - | - |  | Ref. | - | - |  | Ref. | - | - |  | Ref. | - | - |  |
| UUTI | 0.99 | 0.49-2.01 | 0.979 | 0.86 | 0.43-1.72 | 0.667 | **-13.13** | 0.99 | 0.49-2.00 | 0.977 | 0.00 | 0.99 | 0.49-2.01 | 0.979 | 0.00 | 0.97 | 0.48-1.95 | 0.924 | -2.02 |
| Respiratory | 3.73 | 2.05-6.76 | <0.001 | 3.73 | 2.07-6.72 | <0.001 | 0.00 | 3.70 | 2.04-6.69 | <0.001 | -0.80 | 3.70 | 2.04-6.7 | <0.001 | -0.80 | 3.74 | 2.06-6.79 | <0.001 | 0.27 |
| IVD | 0.32 | 0.03-3.24 | 0.335 | 0.28 | 0.03-2.66 | 0.268 | **-12.50** | 0.32 | 0.03-3.26 | 0.339 | 0.00 | 0.30 | 0.03-3.03 | 0.309 | -6.25 | 0.30 | 0.03-3.05 | 0.311 | -6.25 |
| No focus | 0.59 | 0.13-2.65 | 0.489 | 0.48 | 0.11-2.16 | 0.342 | **-18.64** | 0.58 | 0.13-2.62 | 0.480 | -1.69 | 0.59 | 0.13-2.65 | 0.491 | 0.00 | 0.61 | 0.14-2.74 | 0.519 | 3.39 |
| Unknown | 1.38 | 0.76-2.53 | 0.294 | 1.33 | 0.73-2.42 | 0.349 | -3.62 | 1.36 | 0.75-2.49 | 0.312 | -1.45 | 1.37 | 0.75-2.50 | 0.308 | -0.72 | 1.39 | 0.76-2.53 | 0.289 | 0.72 |
| Other | 5.00 | 1.13-22.06 | 0.034 | 3.16 | 0.76-13.19 | 0.115 | **-36.80** | 5.40 | 1.23-23.59 | 0.025 | 8.00 | 5.11 | 1.16-22.38 | 0.031 | 2.20 | 5.03 | 1.14-22.15 | 0.033 | 0.60 |
| Age | 1.04 | 1.02-1.05 | <0.001 | - | - | - |  | 1.04 | 1.02-1.05 | <0.001 | 0.00 | 1.04 | 1.02-1.05 | <0.001 | 0.00 | 1.03 | 1.02-1.05 | <0.001 | -0.96 |
| Case definition |  |  |  |  |  |  |  |  |  |  |  |  |  |  |  |  |  |  |  |
| COCA | 0.99 | 0.62-1.59 | 0.974 | 0.99 | 0.62-1.57 | 0.95 | 0.00 | 0.99 | 0.62-1.59 | 0.982 | 0.00 | 0.99 | 0.62-1.59 | 0.967 | 0.00 | 0.95 | 0.60-1.52 | 0.844 | -4.04 |
| COHA | Ref. | - | - | Ref. | - | - |  | Ref. | - | - |  | Ref. | - | - |  | Ref. | - | - |  |
| HOHA | 1.87 | 1.17-2.97 | 0.009 | 1.84 | 1.16-2.92 | 0.009 | -1.60 | 1.89 | 1.19-3.02 | 0.007 | 1.07 | 1.88 | 1.18-3.00 | 0.008 | 0.53 | 1.85 | 1.16-2.95 | 0.010 | -1.07 |
| Provenance |  |  |  |  |  |  |  |  |  |  |  |  |  |  |  |  |  |  |  |
| Home | Ref. | - | - | Ref. | - | - |  | Ref. | - | - |  | Ref. | - | - |  | Ref. | - | - |  |
| Nursing | 1.59 | 0.94-2.69 | 0.083 | 2.22 | 1.33-3.68 | 0.002 | **39.62** | 1.59 | 0.94-2.69 | 0.082 | 0.00 | 1.63 | 0.97-2.74 | 0.067 | 2.52 | 1.58 | 0.94-2.68 | 0.085 | -0.63 |
| Hospital | 2.46 | 0.94-6.43 | 0.065 | 2.09 | 0.81-5.36 | 0.127 | **-15.04** | 2.57 | 0.99-6.71 | 0.053 | 4.47 | 2.44 | 0.93-6.38 | 0.068 | -0.81 | 2.53 | 0.97-6.61 | 0.057 | 2.85 |
| On dialysis + |  |  |  |  |  |  |  |  |  |  |  |  |  |  |  |  |  |  |  |
| No | Ref. | - | - | Ref. | - | - |  | Ref. | - | - |  | Ref. | - | - |  | Ref. | - | - |  |
| Yes | 3.28 | 1.06-10.14 | 0.04 | 2.89 | 0.97-8.64 | 0.057 | **-11.89** | 3.41 | 1.11-10.46 | 0.032 | 3.96 | 3.21 | 1.04-9.89 | 0.042 | -2.13 | 3.13 | 1.01-9.68 | 0.047 | -4.57 |
| Vascular device* |  |  |  |  |  |  |  |  |  |  |  |  |  |  |  |  |  |  |  |
| No | Ref. | - | - | Ref. | - | - |  | Ref. | - | - |  | Ref. | - | - |  | Ref. | - | - |  |
| Yes | 2.41 | 1.01-5.74 | 0.047 | 2.05 | 0.88-4.74 | 0.095 | **-14.94** | 2.43 | 1.02-5.80 | 0.045 | 0.83 | 2.49 | 1.05-5.89 | 0.037 | 3.32 | 2.45 | 1.03-5.84 | 0.043 | 1.66 |
| Discharge * |  |  |  |  |  |  |  |  |  |  |  |  |  |  |  |  |  |  |  |
| No | Ref. | - | - | Ref. | - | - |  | Ref. | - | - |  | Ref. | - | - |  | Ref. | - | - |  |
| Yes | 1.55 | 1.01-2.38 | 0.047 | 1.54 | 1.01-2.35 | 0.047 | -0.65 | 1.55 | 1.01-2.39 | 0.044 | 0.00 | 1.55 | 1.01-2.38 | 0.046 | 0.00 | 1.54 | 1.01-2.37 | 0.048 | -0.65 |
| Sex |  |  |  |  |  |  |  |  |  |  |  |  |  |  |  |  |  |  |  |
| Male | Ref. | - | - | Ref. | - | - |  | - | - | - |  | Ref. | - | - |  | Ref. | - | - |  |
| Female | 0.8 | 0.55-1.15 | 0.227 | 0.79 | 0.55-1.14 | 0.214 | -1.25 | - | - | - |  | 0.80 | 0.55-1.16 | 0.237 | 0.00 | 0.79 | 0.55-1.14 | 0.210 | -1.25 |
| IMD | 0.98 | 0.92-1.04 | 0.52 | 1.00 | 0.94-1.06 | 0.904 | 2.04 | 0.98 | 0.92-1.04 | 0.553 | 0.00 | - | - | - |  | 0.98 | 0.92-1.04 | 0.537 | 0.00 |
| Neutrophil count + | |  |  |  |  |  |  |  |  |  |  |  |  |  |  |  |  |  |  |
| No | Ref. | - | - | Ref. | - | - |  | Ref. | - | - |  | Ref. | - | - |  | - | - | - | - |
| Yes | 1.48 | 0.75-2.91 | 0.257 | 1.21 | 0.62-2.35 | 0.582 | **-18.24** | 1.51 | 0.77-2.97 | 0.235 | 2.03 | 1.47 | 0.75-2.89 | 0.264 | -0.68 | - | - | - | - |
