# Supplementary material for: Predictive factors of in-hospital mortality in patients with laboratory-confirmed Escherichia coli, Klebsiella species or Pseudomonas aeruginosa bloodstream infections
Source: PLoS One. 2021 Nov 2;16(11):e0259305. doi: 10.1371/journal.pone.0259305 (PMC8562814; doi:10.1371/journal.pone.0259305)
Supplement: S2 Table — VIF; Variance inflation factor, LUTI; Lower urinary tract infection, UUTI; Upper urinary tract infection, IVD; Intravascular device, COCA; Community-onset community associated, COHA; Community-onset healthcare associated, HOHA; Hospital-onset healthcare associated IMD; Index of Multiple Deprivation, + At the time of the GNBSI, * Within <28 days prior to the GNBSI. (DOCX) [file pone.0259305.s002.docx]

|  | **VIF** | **1/VIF** |
| --- | --- | --- |
| Primary focus |  |  |
| Gastrointestinal | 1.14 | 0.875 |
| Hepatobiliary | 1.25 | 0.798 |
| Skin | 1.10 | 0.909 |
| LUTI |  |  |
| UUTI | 1.26 | 0.795 |
| Respiratory | 1.16 | 0.858 |
| IVD | 1.35 | 0.742 |
| no focus | 1.10 | 0.911 |
| Unknown | 1.53 | 0.653 |
| Other | 1.04 | 0.958 |
| Age (years) | 1.16 | 0.862 |
| Case definition |  |  |
| COCA | 1.19 | 0.840 |
| COHA |  |  |
| HOHA | 1.28 | 0.782 |
| Provenance |  |  |
| Home | 1.13 | 0.888 |
| Nursing |  |  |
| Hospital | 1.12 | 0.894 |
| On dialysis + |  |  |
| No |  |  |
| Yes | 1.07 | 0.932 |
| Vascular device <28 days* |  |  |
| No |  |  |
| Yes | 1.58 | 0.631 |
| Discharge <28 days * |  |  |
| No |  |  |
| Yes | 1.04 | 0.963 |
| Sex |  |  |
| Male |  |  |
| Female | 1.03 | 0.972 |
| IMD | 1.05 | 0.948 |
| Neutrophil <500 cells/µL + |  |  |
| No |  |  |
| Yes | 1.07 | 0.933 |

**S2 Table**

LUTI; Lower urinary tract infection, UUTI; Upper urinary tract infection, IVD; Intravascular device, COCA; Community-onset community associated, COHA; Community-onset healthcare associated, HOHA; Hospital-onset healthcare associated, IMD; Index of Multiple Deprivation, + at the time of the GNBSI, *<28 days prior to the GNBSI.
